# Selected Strength Properties of Coal Bottom Ash (CBA) Concrete Containing Fly Ash under Different Curing and Drying Conditions

**DOI:** 10.3390/ma14185381

**Published:** 2021-09-17

**Authors:** Ji-Hun Park, Quang-The Bui, Sang-Hwa Jung, In-Hwan Yang

**Affiliations:** 1 Department of Civil Engineering, Kunsan National University, Kunsan 54150, Korea; jhpark3@kunsan.ac.kr (J.-H.P.); tqbui93@gmail.com (Q.-T.B.); 2 Korea Conformity Laboratories, Seoul 08503, Korea; jsh2593@kcl.re.kr

**Keywords:** coal bottom ash, fly ash, drying conditions, strength properties, ultrasonic pulse velocity

## Abstract

This study aims to evaluate the effect of curing and drying conditions on the strength properties of concrete containing coal bottom ash (CBA) and fly ash as substitutes for fine aggregates and cement, respectively. The strength properties of the concrete including CBA and fly ash were evaluated under two different curing and drying conditions: saturated surface-dry (SSD) conditions and oven-dried conditions at curing ages of 28 and 91 days. The natural fine aggregates of the mixtures were replaced by CBA fine aggregates at 25%, 50%, 75%, and 100% by volume. In addition, the cement in the mixtures was partly replaced with fly ash at 20% and 40%. The experimental program included the measurement of the unit weight, compressive strength, splitting tensile strength, flexural strength, and ultrasonic pulse velocity of the concrete. The test results showed that the compressive strength, splitting tensile strength, and flexural strength decreased as the CBA content increased under both SSD and oven-dried conditions. The curing and drying conditions of the concrete with CBA and fly ash considerably influenced the reduction in the compressive, splitting, and flexural tensile strengths of the concrete. Additionally, the experimental results showed that fly ash insignificantly contributed to the reduction in the strength properties under both SSD and oven-dried conditions. Finally, the relationships between ultrasonic pulse velocity and the splitting tensile strength, flexural tensile strength, and compressive strength were investigated.

## 1. Introduction

Sustainable development in the construction industry is pursued by many nations worldwide, so the utilization of waste from the industry has become more essential. Moreover, the utilization of waste as a binder in concrete can reduce the amount of cement used, which ultimately reduces the price of the concrete and the carbon dioxide emissions from the cement industry [1]. Therefore, several studies have been conducted to investigate the utilization of waste materials in concrete [2,3,4,5]. Among these waste materials, coal bottom ash (CBA) and fly ash are increasingly being released from thermal power plants. Due to the development of concrete technology, CBA and fly ash can currently be utilized as construction materials in concrete [6,7,8,9,10]. Specifically, bottom ash with different levels of fineness was used as the replacement of cement in a study by Abdulmatin et al. [6]. Gollakota et al. [7] reviewed the applications of fly ash in construction materials and its potential in geopolymer concrete. Singh et al. [8] analyzed the characteristics of fly ash in self-compacting concrete (SCC). The utilization of CBA as a partial cement replacement has significant potential in improving the strength, durability, and microstructure of concrete [9]. Oruji et al. [10] investigated the replacement ability of cement by ultrafine CBA. Moreover, Menéndez et al. [11] evaluated the pozzolanic activity of a novel cement including CBA and fly ash.

The utilization of CBA as a fine aggregate for fabricating concrete has been reported by several studies [12,13,14,15,16]. According to the study of Singh and Siddique [14], the pozzolanic reaction of CBA improved the compressive strength of CBA concrete during extended curing, and CBA concrete exhibited a better resistance to sulfuric acid attack than conventional concrete. Singh et al. [17] also investigated the material properties of SCC containing CBA and recycled coarse aggregates. The experimental results indicated that the strength properties of the SCC were enhanced to a certain degree by the presence of CBA. The study of Singh and Siddique [18] estimated the influence of the CBA content on the workability and strength properties of CBA concrete. In this study, the CBA concrete exhibited compressive and splitting tensile strengths similar to those of the reference concrete at the long-term curing age. However, according to some studies [6,14,19], the strength properties of concrete containing CBA as a replacement for natural fine aggregates were worse than those of the reference concrete at short-term curing ages of 7 and 28 days. 

Fly ash has been applied in concrete as a cementitious material or filler material. It is known that the use of fly ash in concrete has many benefits, such as reducing the hydration heat, increasing the workability of fresh concrete, reducing the shrinkage, and especially improving the strength of concrete at long-term curing ages [20,21,22,23]. Due to the pozzolanic properties of fly ash, it improved the durability of the concrete [20]. Additionally, the inclusion of fly ash in concrete mixtures reduced the hydration rate and shrinkage rate in the concrete [21]. Sahmaran and Li [22] showed that the tensile strain capacity of cementitious composite specimens with high volumes of fly ash was improved at a long-term curing age. Tran et al. [23] extensively reviewed mitigation strategies of drying shrinkage in cementitious materials. They suggested that replacing cement with supplementary cementitious materials such as fly ash was an effective approach to mitigate drying shrinkage. In addition, according to the study by Bentz and Ferraris [24], the presence of fly ash in concrete mixtures extended the setting time of fresh concrete. Durán-Herrera et al. [25] concluded that fly ash significantly reduced the heat release peak temperature at early curing ages and thus extended the setting time of concrete. This conclusion was also recognized in the study by Nath and Sarker [26]. They insisted that the extent of improvement of high strength concrete was due to the contributions of fly ash. ACI 211 [27] recommended that the substitution of cement with fly ash should be in the range of 10% to 35% to obtain advantages from the use of fly ash. Although the presence of fly ash improved the durability and long-term strength of concrete, the replacement of cement with fly ash led to a reduction in the early strength of concrete in several studies [20,28,29].

Although CBA and fly ash have been widely applied in the concrete industry, few studies [30,31] have been conducted to investigate the combined effects of CBA and fly ash on the material properties of concrete. Majhi and Nayak [30] found that the strength properties of the mixture, which combined 30% CBA and 20% fly ash to replace sand and cement, respectively, were greater than those of the other mixtures. Rafieizonooz et al. [31] carried out the study by combining 0, 25, 50, 75, and 100% bottom ash as fine aggregate and 20% fly ash as a binder in concrete mixtures. The results reflected that the strength properties of the concrete combined with CBA and fly ash were similar to or better than those of the reference concrete. Moreover, fly ash and CBA have high water-absorption capacities, and therefore, the moisture contents or drying conditions in concrete can affect the material properties of concrete. Papayianni and Valliasis [32] investigated heat deformations of fly ash concrete and concluded that concrete including a high volume of fly ash was more sensitive to heating. Several studies on conventional concrete [33,34,35] indicate that there are various effects on its properties due to the moisture content in concrete. However, there are few studies on the effect of drying conditions on the strength properties of concrete containing CBA and fly ash.

Therefore, this study aimed to investigate the effect of curing and drying conditions on the strength properties of concrete containing CBA and fly ash. The mechanical properties of concrete were evaluated under two different drying conditions: saturated surface-dry (SSD) conditions and oven-dried conditions. In addition, the effects of the CBA and fly ash contents on the mechanical properties of the concrete were analyzed. The strength properties of the concrete with CBA and fly ash at each drying condition were measured at curing ages of 28 and 91 days. Finally, the relationships between the splitting tensile strength, flexural tensile strength, compressive strength, and ultrasonic pulse velocity were provided based on the test results.

## 2. Material and Mixing Proportions

### 2.1. Materials

**Figure 1:**
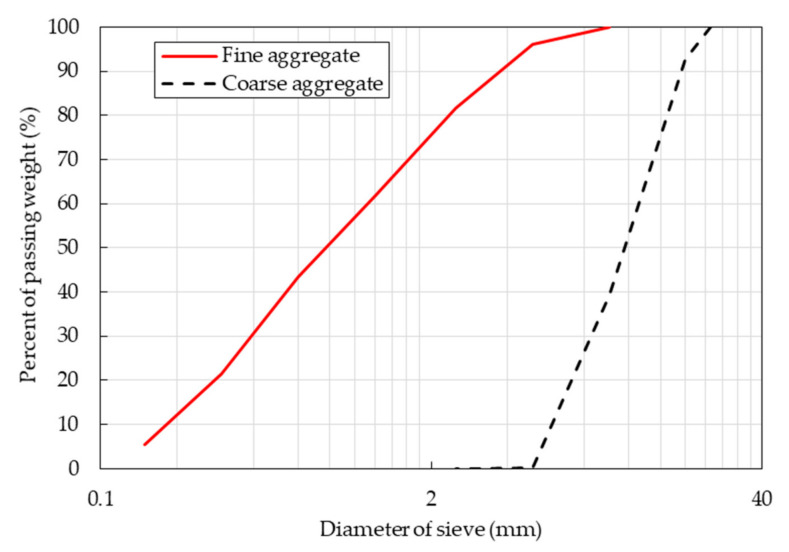
Sieve analysis results of the natural coarse and fine aggregates.

The natural coarse and fine aggregates used in this study were crushed. Both natural coarse and fine aggregates were tested to determine their physical properties, including their density, water-absorption capacity, and particle size distribution. The density of aggregates was measured under SSD conditions. The SSD condition was considered the state in which the aggregate had a dry surface but the particle pores in aggregates were saturated with water. Thus, the water absorption from the aggregate could be disregarded. The physical properties of the natural coarse and fine aggregates are presented in Table 1. The densities of the natural coarse and fine aggregates were similar at 2.60 and 2.61 g/cm^3^, respectively. The natural fine and coarse aggregates had maximum sizes of 5 and 20 mm, respectively. The particle size distributions of the natural coarse and fine aggregates are shown in Figure 1.

The CBA and fly ash used in this study were collected from a thermal power plant company (Korea South-East Power Co., Ltd., Yeongheung Power Division, Yeongheung, Korea). CBA was used as a substitute for natural fine aggregates in the mixtures. The test results in Table 1 show that the density of CBA, at 1.84 g/cm^3^, was lower than that of the natural fine aggregate. The water absorption rate of fine CBA aggregate is the rate of the increase in mass due to water in the pores of the fine aggregate. The water absorption of aggregate would increase with the internal pore structures of aggregate. Generally, the number of pores in fine CBA aggregates is greater than that in fine and coarse natural aggregates. Therefore, the water absorption of the CBA fine aggregate used in this study was much greater than that of the natural fine and coarse aggregates. Specifically, the water absorption of CBA, at 3.88%, was approximately six times greater than that of the natural fine aggregate, at 0.64%.

**Figure 2:**
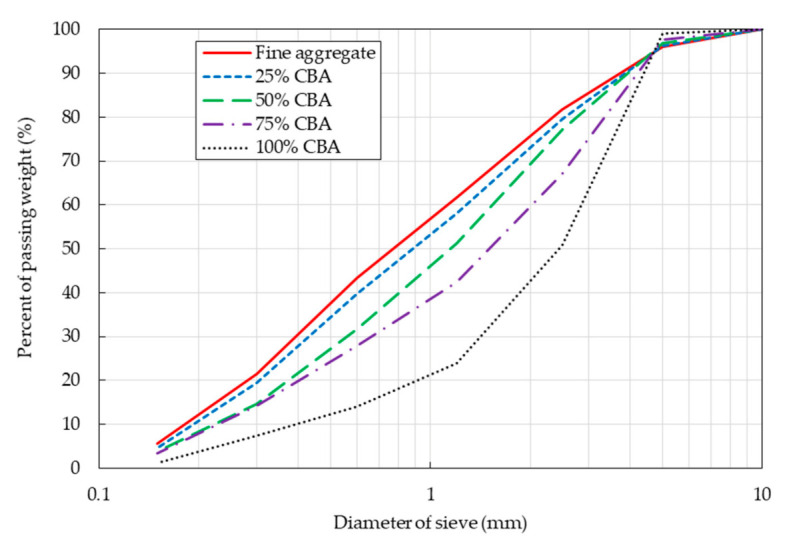
Sieve analysis of the combinations with CBA and natural fine aggregate at different CBA contents.

To make the maximum size of the CBA similar to that of the fine aggregate, the original CBA obtained from a thermal power plant was ground with a ball mill. Then, CBA with particle sizes in the range of 0.15~5.0 mm was chosen to replace the fine aggregates by the sieve test. The sieve analysis results of the combinations of CBA and natural fine aggregate at different replacement ratios are shown in Figure 2. The figure shows that the particle size distribution of the fine aggregate combination depended on the CBA content.

By using an X-ray fluorescence (XRF) spectrometer, the chemical compositions of CBA and fine aggregates were obtained, as shown in Table 2. The results indicated that the CBA used was a silicon-rich material with higher silicon dioxide (SiO_2_) occupying more than 50% of the total amounts of components. The other main components of the CBA aggregate were Al_2_O_3_, Fe_2_O_3_, and CaO.

The binder used in this study included ordinary Portland cement (OPC) and fly ash. OPC was supplied by Ssangyong C&E Company (Ssangyong C&E Co., Jung-gu, Seoul, Korea). The OPC used in the mixtures was type I with an initial setting time of 45 min and a final setting time of 360 min. The density and fineness modulus of the OPC were 3.15 g/cm^3^ and 2800 cm^2^/g, respectively. Additionally, fly ash had a density and fineness modulus of 2.23 g/cm^3^ and 3650 cm^2^/g, respectively. The chemical compositions of OPC and fly ash are shown in Table 2. This analytical result shows that both fly ash and CBA consisted of large amounts of SiO_2_ and Al_2_O_3_.

### 2.2. Mixing Proportions

To investigate the effects of fly ash and CBA on the properties of the concrete, two series of mixing proportions in terms of fly ash contents were prepared. The mixing proportions of this study are presented in detail in Table 3. Each mixture is identified by the replacement percentage of cement by fly ash and by that of natural fine aggregate by CBA.

For the first series of mixing proportions, the cement was replaced by fly ash at a replacement ratio of 20% by volume, and the natural fine aggregates were replaced by CBA at four different replacement ratios by volume: 25%, 50%, 75%, and 100%. For the second series of mixing proportions, the volume of cement was replaced by fly ash at a replacement ratio of 40%, and the volume of natural fine aggregates was replaced by CBA at two different replacement ratios: 50% and 100%. The last two digits in the mixture identifications represented the curing age of the concrete specimens.

The water to binder ratios of the first and second mixing series were 0.32 and 0.34, respectively.

The water-absorption capacity of CBA was much greater than that of natural fine aggregates. Thus, in particular, the aggregates and CBA were prepared under SSD conditions to ensure that they did not absorb the mixing water. The ratio of fine aggregate content to total aggregate content remained constant at 0.43 by volume. A high-range water-reducing agent (HWRA) and air-entraining (AE) admixture were added to the mixture to improve the workability of the concrete mixtures. The HWRA and AE were supplied by Silkroad C&T Company (Silkroad Co., Gangnam-gu, Seoul, Korea). The amount of HWRA was equivalent to 1.2% binder by weight, while that of the AE admixture was equal to 2.8% HWRA by weight.

## 3. Experimental Methods

### 3.1. Casting and Curing of Specimens

Cylindrical specimens with dimensions of 100 mm (diameter) × 200 mm (length) were cast to measure the mechanical properties of CBA concrete, including the unit weight, compressive strength, and splitting tensile strength. Prism specimens with dimensions of 100 mm × 100 mm × 400 mm were cast to measure the flexural tensile strength. To investigate the effects of curing and drying conditions on the mechanical properties, two different curing and drying conditions for concrete specimens were considered: SSD conditions and oven-dried conditions.

For the SSD condition, the specimens were demolded after 24 h of concrete casting and then cured in a water tank at 24 ± 2 °C until the testing date. Thereafter, the moisture on the concrete specimen surface was removed with towels before testing. Additionally, for the oven-dried conditions, the specimens were demolded after 24 h of concrete casting and then cured in a water tank at 24 ± 2 °C for 7 days. After that, they were cured at room temperature in the laboratory until 1 day before testing. Finally, the concrete specimens were dried in an oven at 105 ± 5 °C for 24 h before the measurement.

CBA is a high water-absorption material, and thus, moisture content or drying conditions can strongly affect the properties of concrete, including CBA. Typical curing of concrete at room temperature is an actual condition for concrete structures in construction sites. However, the moisture content of concrete specimens under typical conditions depends on the room temperature and humidity. The room temperature and humidity also vary with the environmental conditions under which the test is performed. Consequently, the test results at room temperature may vary depending on the temperature and humidity to which the concrete specimen is subjected. Therefore, the method of concrete curing at room temperature is not considered in this study.

Meanwhile, the SSD condition is an ideal condition under which the concrete specimen is fully saturated with moisture. Oven-drying conditions are another ideal condition under which concrete specimens are assumed to be dried completely. In terms of moisture contents and drying conditions, the SSD conditions and oven-dried conditions selected in this study are two extreme conditions because actual drying conditions of concrete specimens probably exist on the spectrum between SSD conditions and oven-dried conditions. Therefore, test results obtained under the SSD and oven-dried conditions might be meaningful in academic research, although they do not reflect the actual drying conditions in practice.

### 3.2. Testing Procedure

For the fresh concrete, the workability of each mixture was examined by implementing a slump test according to Korean standard KS F 2402 [36]. For hardened concrete, the effects of the drying conditions on the material properties of the concrete were investigated.

The material properties of the F20 series specimens in Table 3 were measured at curing ages of 28 and 91 days, considering the two different curing and drying conditions. Additionally, the mechanical properties of the F40 series specimens in Table 3 were measured at only 28 days, also considering the two different curing and drying conditions.

**Figure 3:**
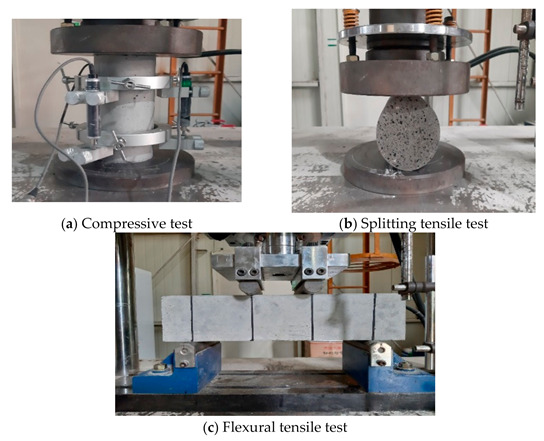
Material test setup.

To investigate the material properties, the unit weight, compressive strength, and splitting tensile strength of the CBA concrete were measured by using cylindrical specimens in accordance with Korean standards KS F 2405 and KS F 2423 [37,38]. According to KS F 2405, at least three cylindrical specimens with dimensions of 100 mm (diameter) × 200 mm (length) are required to perform the compressive test. To obtain the displacement of the test specimen, three linear displacement transducers (LVDTs) were attached around the specimen, as shown in Figure 3a. The load-deflection curves, which were obtained from the test, were calculated to obtain the stress–strain curves, and then the elastic modulus of each specimen was estimated. For the splitting tensile test, at least three cylindrical specimens with dimensions of 100 mm × 200 mm are also required. The test setup of the splitting tensile test is shown in Figure 3b. To measure the flexural tensile strength, prism specimens were used in accordance with Korean standard KS F 2408 [39]. The prismatic specimens were tested under four-point loading, as shown in Figure 3c. For each material property, five specimens were measured, and the mean and standard deviation of the measurements were represented in the test results.

Finally, the ultrasonic pulse velocity was measured by using transducers and a pulse generation circuit with a frequency of 54 kHz in accordance with ASTM C597-02 [40]. Before the experiment was performed, the cylindrical concrete specimens were polished on both ends. The velocity of the pulses transferred through concrete specimens by the transducers was analyzed.

## 4. Results and Discussion

### 4.1. Workability

**Figure 4:**
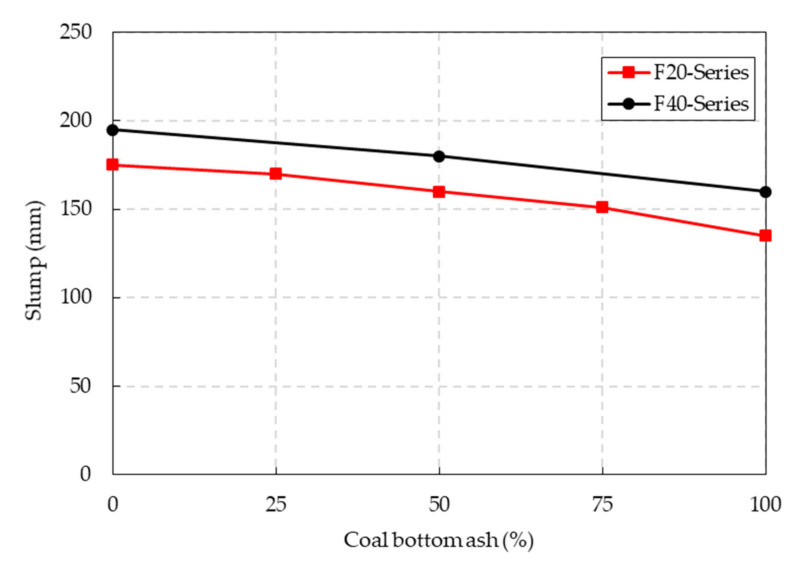
Slump test results of the CBA concrete.

The slump test results of the F20 and F40 series specimens are shown in Figure 4. The slump of the concrete mixture decreased gradually as the CBA contents in the mixture increased. For the F20 series specimens, the slump of the concrete mixture decreased from 175 mm to 135 mm when the CBA contents increased from 0 to 100%. Similarly, the F40 series specimens exhibited a decreasing trend in slump values, varying from 195 mm to 160 mm. According to the study by Singh and Siddique [12], a decrease in the slump in CBA concrete was also observed as the CBA content increased. The decrease in the slump resulted from the higher specific surface area of the CBA fine aggregate compared to that of the natural fine aggregate. Additionally, the irregular shapes of the CBA contributed considerably to the decrease in the slump of the mixture because the irregular shape and rough texture of the CBA increased the friction between the particles in the mixture.

The slump of the F40 mixture series was greater than that of the F20 mixture series. At the different CBA contents, an increase of approximately 15% in slump was observed in the F40 mixture series. This meant that the fly ash contents improved the workability of the CBA concrete mixture. The increase in slump of the F40 mixtures was due to the characteristics of the fly ash particles. According to scanning electron microscopy (SEM) results, the fly ash particles had a smooth surface, a spherical shape, and a small size compared to OPC particles. These characteristics effectively reduced interparticle friction and created a ball-bearing effect in the particle matrix of the mixture.

### 4.2. Unit Weight

**Figure 5:**
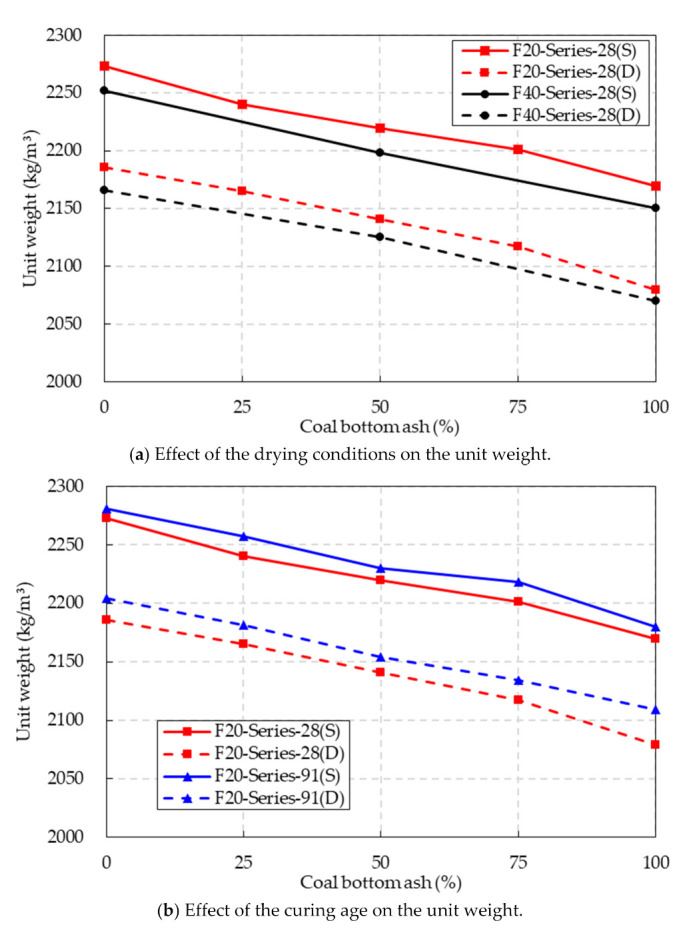
Unit weight test results.

The unit weight test results of the F20 and F40 series under the SSD conditions and oven-dried conditions are presented in Figure 5a. In the figure, the characteristics of S and D in parentheses in mixture series identification indicate the SSD condition and oven-dried condition, respectively.

These results were obtained at a curing age of 28 days. The results indicated that higher CBA contents in the concrete specimens led to a reduction in the unit weight. For the SSD condition, the unit weight of the F20 and F40 series decreased by 4.5% and 4.7%, respectively, when the CBA contents increased from 0% to 100%. It was assumed that compared to the density of fine natural aggregate, the low density of the CBA fine aggregate was the main reason for the decrease in the unit weight in the CBA concrete [12].

For the oven-dried conditions, a reduction in the unit weight was also observed in both the F20 and F40 series. The unit weight of the F20 series decreased by 5.1% and that of the F40 series decreased by 4.5%. The decrease in the unit weight in the CBA concrete was due to the porosity of the CBA fine aggregate being higher than that of the natural fine aggregate.

The test results also indicated that the drying condition affected the unit weight of the CBA concrete. The unit weights of specimens in the oven-dried conditions were lower than those of specimens under the SSD conditions. For the F20 series, specifically, the unit weight of the specimens under the oven-dried conditions at the various CBA contents was 3.4~4.2% lower than that under the SSD conditions. For the F40 series, the unit weight of the specimens under the oven-dried conditions at the various CBA contents was 3.4~3.8% lower than that under the SSD conditions.

In addition, the fly ash contents affected the unit weight of the CBA concrete. For both SSD and oven-dried conditions, the unit weight of the CBA concrete with a high fly ash content was lower than that of the CBA concrete with a low fly ash content. The density of the fly ash was lower than that of OPC; therefore, replacement of the OPC by fly ash contributed to the reduction in the unit weight of the CBA concrete.

Figure 5b shows a comparison of the unit weights of the F20 series specimens at curing ages of 28 and 91 days. The test results showed that the unit weights of the concrete specimens increased slightly as the curing age increased from 28 to 91 days under both SSD and oven-dried conditions.

### 4.3. Compressive Strength and Elastic Modulus

**Figure 6:**
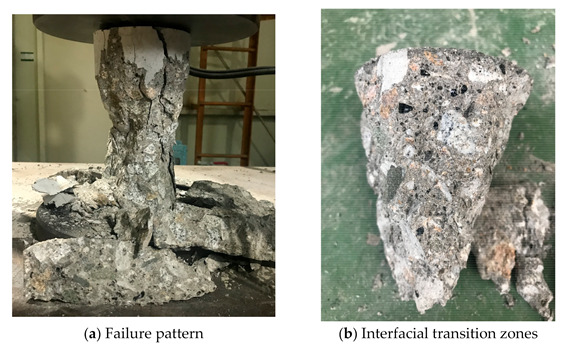
Typical compressive failure pattern of the CBA concrete.

The typical failure pattern of the specimen under the compressive strength test is presented in Figure 6. A conical shape of the specimen was observed at failure, as shown in Figure 6b. The failed surface showed that the interfacial transition zones between natural coarse aggregates and cement paste were damaged, and some cracks passed through the coarse aggregates. Meanwhile, many cracks passed through CBA aggregates. Observation of the damage of the sample implied that high substitution of fine aggregate by CBA might contribute to crack initiation and development through the CBA aggregate and finally result in the strength reduction of the CBA concrete.

**Figure 7:**
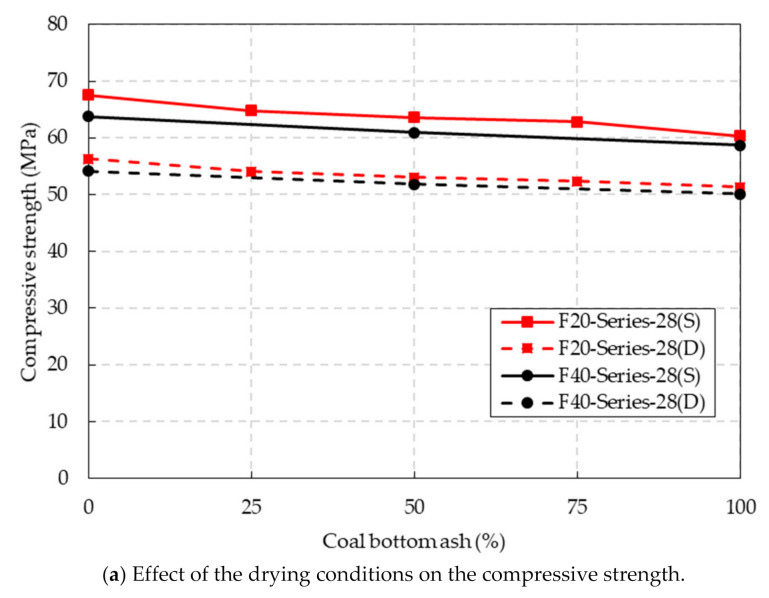

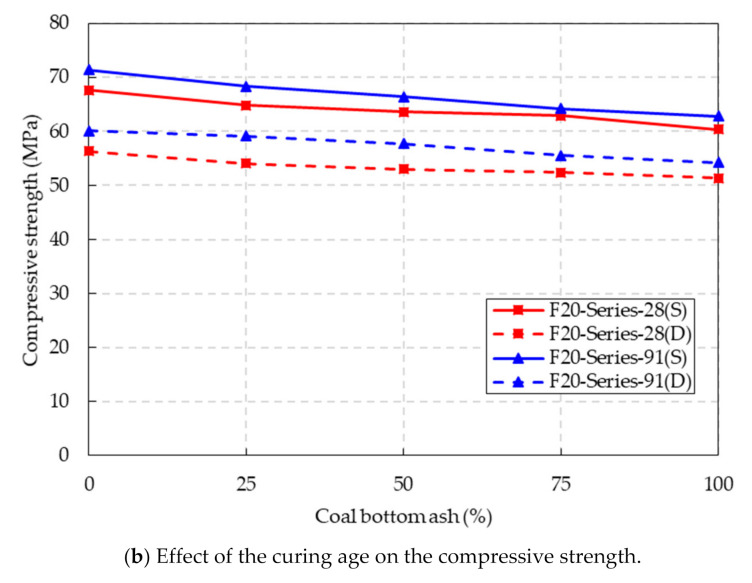
Compressive strength test results.

The compressive strength test results of the CBA concrete under the two different drying conditions are presented in Figure 7a. Overall, the compressive strength of the CBA concrete was slightly affected by the CBA content. The compressive strengths of both the F20 and F40 series specimens under SSD and oven-dried conditions decreased slightly as the CBA content increased from 0% to 100%.

A slight decrease in the compressive strength was also reported by Rafieizonooz et al. [31]. Moreover, the study of Cheriaf et al. [41] showed that the acceleration of the pozzolanic reaction of CBA at a curing age of more than 28 days was remarkable and that the increase in the compressive strength due to the pozzolanic reaction would overcome part of the compressive strength decrease in the concrete containing CBA as the replacement of fine aggregate. In particular, for the oven-dried conditions, the compressive strengths of the F20 and F40 series decreased by 8.9% and 7.4%,

The effect of the drying conditions of the specimen on the compressive strength was significant. After the CBA concrete specimens were oven-dried, the compressive strength of the concrete specimens was 15.1~16.7% lower for the F20 series and 14.7~15.2% lower for the F40 series at various CBA contents. According to the study by Hager [42], hydrated calcium silicate (C-S-H gel) is dehydrated at 105°C, which partially damages the cement paste structure. This phenomenon might cause the compressive strength of the CBA concrete to decrease. Additionally, the porosity of the concrete increased due to water evaporation as the temperature increased. This also led to a decrease in the compressive strength of the CBA concrete.

The replacement of cement with fly ash tended to decrease the compressive strength of the CBA concrete. As an example, the compressive strength of the F40-B050–28(S) specimen was 4.3% lower than that of the F20-B025–28(S) specimen under SSD conditions. The difference between the compressive strengths of the F20 and F40 series in oven-dried conditions was not significant. As an example, the compressive strength of the F20-B050–28(D) specimen was 53.0 MPa while that of the F40-B050–28(D) was 51.9 MPa.

The effect of the curing age on the compressive strength of the F20 series is shown in Figure 7b. The test results indicated that a longer curing age improved the compressive strength of the CBA concrete. The compressive strength increased by 2.1~5.3% under the SSD conditions and by 5.3~8.6% under the oven-dried conditions as the curing age of CBA concrete increased from 28 to 91 days. Fly ash and CBA are pozzolanic materials, and pozzolanic reactions also develop over long curing ages. As shown in Table 2, the fly ash and CBA used in this study contained large amounts of SiO_2_ and Al_2_O_3_. These compositions played an important role in the pozzolanic reaction. Therefore, the development of the pozzolanic reaction with curing age produced calcium silicates and aluminates, which filled the voids in the concrete matrix and improved the strength of the concrete.

In addition, the curing-time extension motivates the complete hydration of the cementitious materials, which contributes to improving the strengths of concrete at long curing ages. Thus, a greater compressive strength was observed at 91 days of curing due to the combined effects of the curing age and pozzolanic reaction of the substituted materials. This phenomenon was also reported in previous studies [28,29].

**Figure 8:**
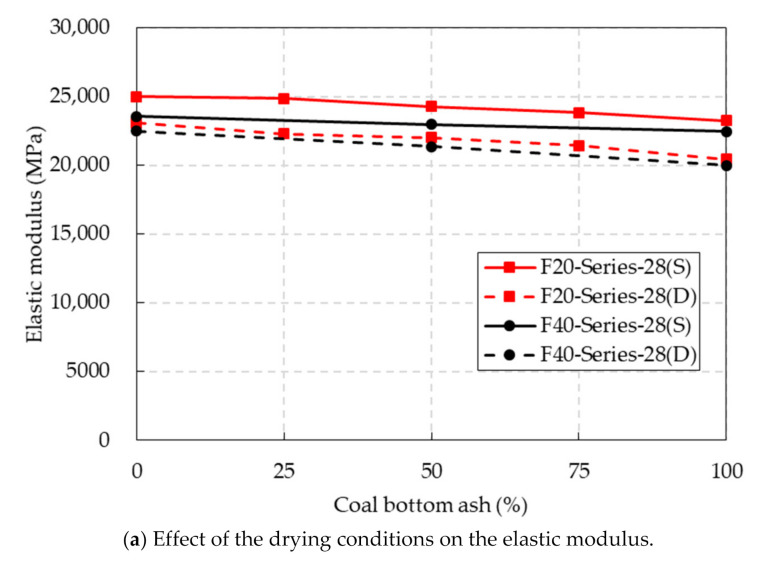

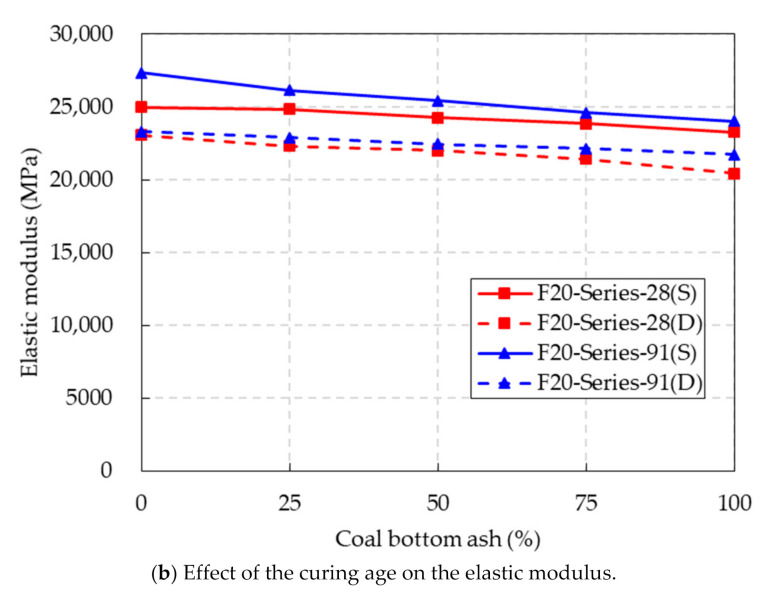
Elastic modulus test results.

The elastic modulus of the concrete mixture is provided in Table 4. The elastic modulus of the CBA concrete was also affected by the drying conditions and curing ages, as shown in Figure 8. The elastic moduli of the F20 and F40 series mixtures under oven-dried conditions were slightly lower than those under SSD conditions. The elastic moduli of the F20 series mixtures at a curing age of 91 days were slightly greater than those at a curing age of 28 days. The test results indicated that the elastic modulus of the CBA concrete increased with curing age.

### 4.4. Splitting Tensile Strength

**Figure 9:**
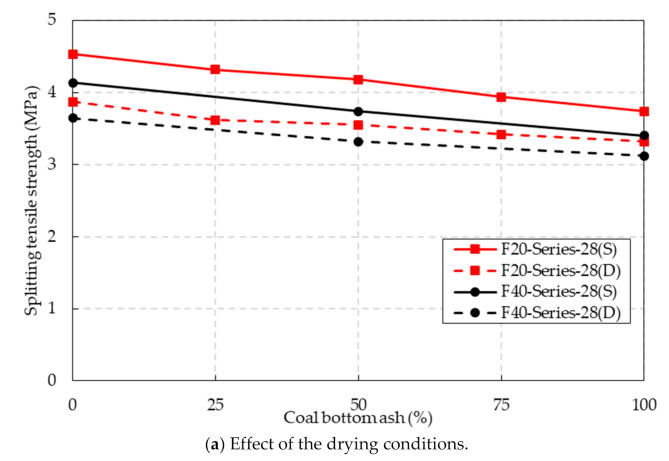

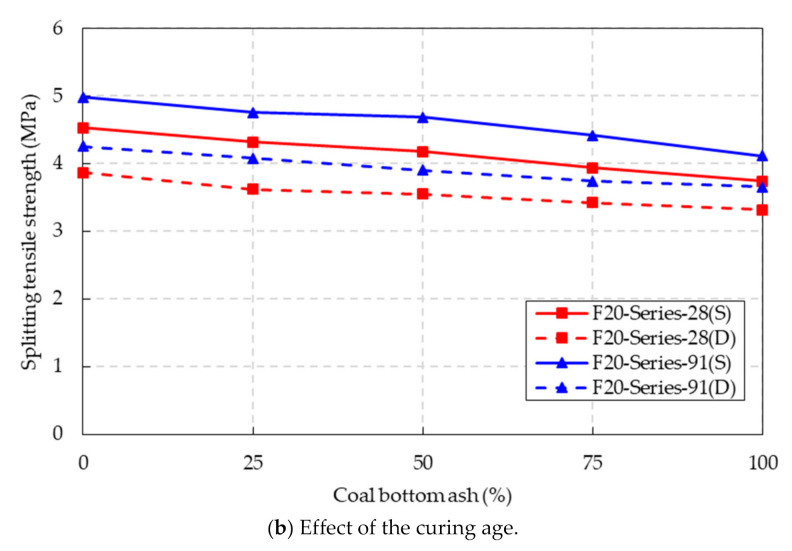
Splitting tensile strength test results.

The splitting tensile test results of the CBA concrete are provided in Table 5. Figure 9a provides the splitting tensile strength of the CBA concrete under the SSD and oven-dried conditions at 28 days of curing age. The test results indicated that the splitting tensile strengths of the CBA concrete decreased considerably as the CBA content increased. The splitting tensile strengths of the F20 and F40 series specimens under the SSD conditions decreased by approximately 17.4% and 17.9%, respectively, as the CBA contents increased from 0% to 100%. However, in the study by Singh and Siddique [12], the splitting tensile strength of the mixture with 25% CBA content showed the greatest value; thus, a gradual decrease in splitting tensile strength was not observed at a curing age of 28 days. CBA particles have a more porous structure than natural fine aggregate particles, which leads to a reduction in the CBA density. In addition, internal cracks easily develop due to the porous structure of the CBA particles when load is applied to the CBA concrete. Therefore, the strength of the CBA concrete decreased as the CBA content increased.

The drying condition also had a significant influence on the splitting tensile strength of the CBA concrete. The splitting tensile strength under the oven-dried conditions was lower than that under the SSD conditions. The reduction rate in the splitting tensile strength of the F20 series ranged from 11.2% to 16.2%, while that in the splitting tensile strength of the F40 series ranged from 8.2% to 11.8%. The evaporation of free water in cement paste and water in the C-S-H gel also contributed to the reduction in the splitting tensile strength of the CBA concrete.

The influence of the fly ash content on the splitting tensile strength can also be observed in Figure 9a. The CBA specimen containing a high fly ash content exhibited a low splitting tensile strength at the same CBA content. For the SSD conditions, the splitting tensile strengths of the F40-B050–28(S) and F40-B100–28(S) specimens were 3.7 and 3.4 MPa, which were 15.0% and 9.1% lower than those of the F20-B050–28(S) and F20-B100–28(S) specimens, respectively. For the oven-dried conditions, the splitting tensile strengths of the F40-B050–28(D) and F40-B100–28(D) specimens were 6.5% and 6.0% lower than those of the F20-B050–28(D) and F20-B100–28(D) specimens, respectively.

The influence of the curing age on the splitting tensile strength of the F20 series is shown in Figure 9b. The test results indicated that the splitting tensile strength of the F20 series was improved by extending the curing age from 28 to 91 days. Under the SSD conditions, the splitting tensile strengths of the F20-series-91(S) specimens were 10.0~12.0% higher than those of the F20-series-28(S) specimens. Under the oven-dried conditions, the splitting tensile strengths of the F20-series-91(D) specimens were 9.4~12.7% higher than those of the F20-series-28(D) specimens. The continuation of the hydration at the long-term curing age and the pozzolanic reaction of the CBA and fly ash enhanced the splitting tensile strength of the CBA concrete.

The tensile strength of concrete can be determined by either a direct tensile test or an indirect tensile test. In the direct tensile test method, there is difficulty gripping both ends of a concrete specimen, and eccentricity at both ends of a specimen can cause variability in the experimental tensile strength results. Therefore, the indirect tensile test method is preferred. The indirect tensile test methods include splitting and flexural tensile tests, which were used in this study. In particular, the flexural tensile strength, or modulus of rupture of concrete, can be used to control tensile cracks in concrete members.

According to ACI 363R-10 [43], the direct tensile strength of concrete can be estimated by using the splitting tensile strength, as expressed in the following equation:

(1) $$f^{\prime }{}_{t}=\;0.42\;\;\mathrm{to}\;\;0.98\sqrt{f_{t}^{}}\;\;$$

The direct tensile strength of concrete can also be estimated by using the flexural tensile strength, as expressed in the following equation:

(2) $$f^{\prime }{}_{t}=\;0.27\;\mathrm{to}\;\;0.62\sqrt{f_{r}^{}}\;\;$$

To determine the precise coefficient of the equation, it is necessary to perform the direct tensile test and analysis of the test results. Estimating the coefficient of the equations will be conducted in future studies.

### 4.5. Flexural Tensile Strength

**Figure 10:**
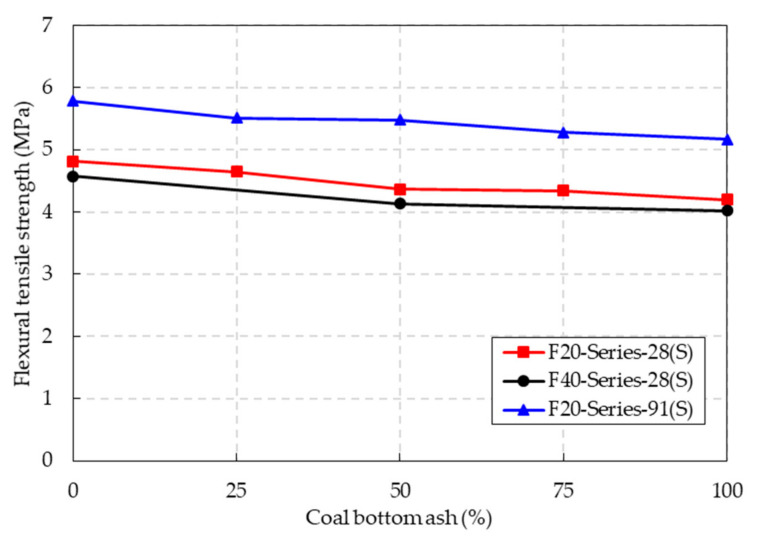
Flexural tensile strength of the CBA concrete.

The flexural tensile strength results of the CBA concrete under the SSD conditions are shown in Figure 10 and Table 5. As in the case of the compressive and splitting tensile strengths, the flexural tensile strength decreased as the CBA content increased. Specifically, compared to that of the control specimens, the splitting tensile strengths of the F20 series specimens containing 25%, 50%, 75%, and 100% CBA were 3.5%, 9.4%, 9.9%, and 12.8% lower, respectively. Additionally, compared to that of the control specimens, the flexural tensile strengths of the F40 series specimens containing 50% and 100% CBA contents were 9.6% and 12.2% lower, respectively.

The flexural tensile strength of the F20 series specimens at a curing age of 91 days is shown in Figure 10. For the series specimens, the flexural tensile strengths increased significantly as the curing age increased from 28 to 91 days. When the CBA contents of the F20 series concrete specimens were 25%, 50%, 75%, and 100%, the flexural tensile strengths of the concrete specimens at a curing age of 91 days were 18.7%, 25.6%, 21.7%, and 23.1% higher than those of the concrete specimens at a curing age of 28 days, respectively. This implied that the pozzolanic reactions due to CBA improved the flexural tensile strength with long-term curing age. The effect of long-term curing age also improved the hydration level of the cementitious material and finally enhanced the flexural tensile strength of the CBA concrete. The results in this study are in good agreement with the results of a study by Saha [28].

### 4.6. Relationships between the Compressive Strength, Splitting Tensile Strength and Flexural Tensile Strength

**Figure 11:**
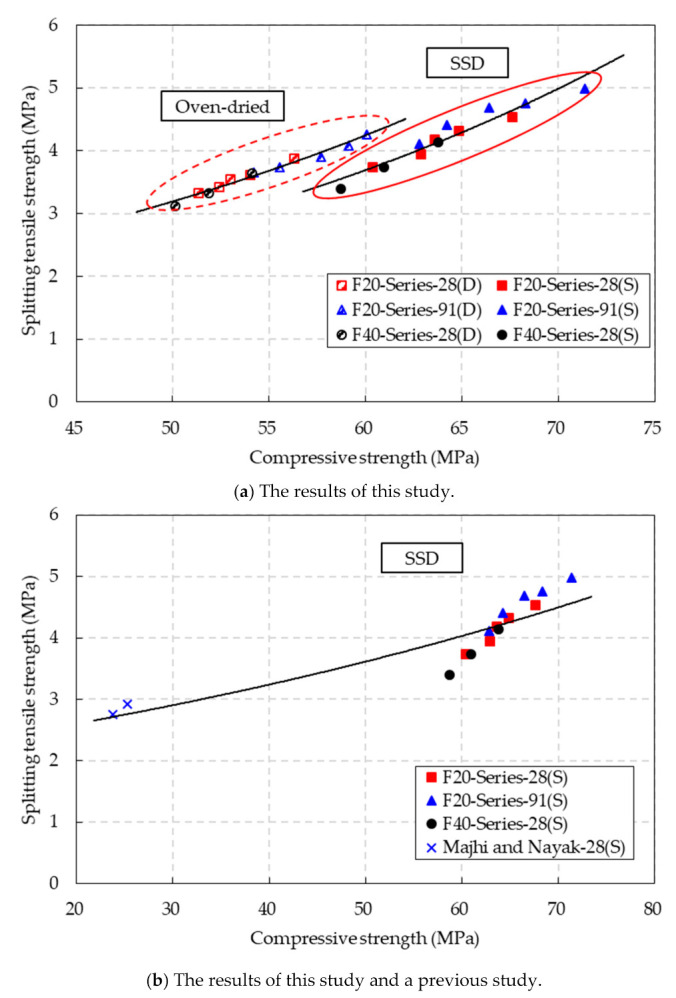
Relationship between the splitting tensile strength and compressive strength of the CBA concrete.

The relationship between the splitting tensile strength and compressive strength under the SSD and oven-dried conditions is shown in Figure 11a. The test results show that the splitting tensile strength increased with the compressive strength of the CBA concrete under both the SSD and oven-dried conditions. By regression analysis, the relations between the splitting tensile strength and compressive strength under two different aging conditions were expressed exponentially by the following equations.

(3) $$f_{t,\mathrm{OD}}=0.767e^{0.0285f_{c,\mathrm{OD}}^{\prime }}\;R^{2}=0.9724\;(\mathrm{Oven}-\mathrm{dried}\;\mathrm{conditions})$$

(4) $$f_{t,\mathrm{SSD}}=0.6064e^{0.0301f_{c,\mathrm{SSD}}^{\prime }}\;R^{2}=0.9253\;(\mathrm{SSD}\;\mathrm{conditions})$$

where *f_t,_*_OD_ and *f_t,_*_SSD_ are the splitting tensile strength (MPa) under the oven-dried and SSD conditions, respectively. *f′_c,_*_OD_ and *f′_c,_*_SSD_ are the compressive strengths (MPa) under oven-dried and SSD conditions, respectively. The coefficients of determination (*R*^2^) of this equation were close to 1, which indicates that these equations could be used to precisely predict the values of the splitting tensile strength by using the compressive strength of CBA concrete.

In addition, based on the test results in this study and in another study [30], the relationship between the splitting tensile strength and compressive strength of the CBA concrete is presented in Figure 11b. There were few data from the other study. The proposed equation for predicting the relationship between the splitting tensile strength and compressive strength of CBA concrete is shown as follows:

(5) $$f_{t,\mathrm{SSD}}=2.0914e^{0.0109f_{c,\mathrm{SSD}}^{\prime }}\;R^{2}=0.8365\;(\mathrm{SSD}\;\mathrm{conditions})$$

where *f_t,_*_SSD_ is the splitting tensile strength (MPa) and *f′_c,_*_SSD_ is the compressive strength (MPa) under the SSD conditions.

Overall, the prediction is in good agreement with the test results. Specifically, the equation could more precisely predict the splitting tensile strength of the CBA concrete with low fly ash (F20 series), while it overestimated the splitting tensile strength of the CBA concrete with high fly ash (F40 series). In addition, the coefficient of determination for this equation is high, at a value of 0.8365. This implies that the splitting tensile strength could be predicted practically by using the proposed equation.

**Figure 12:**
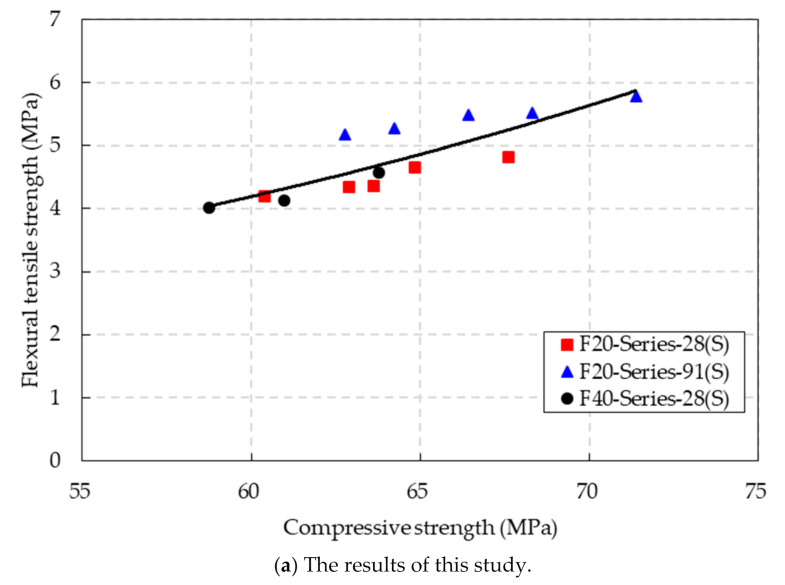

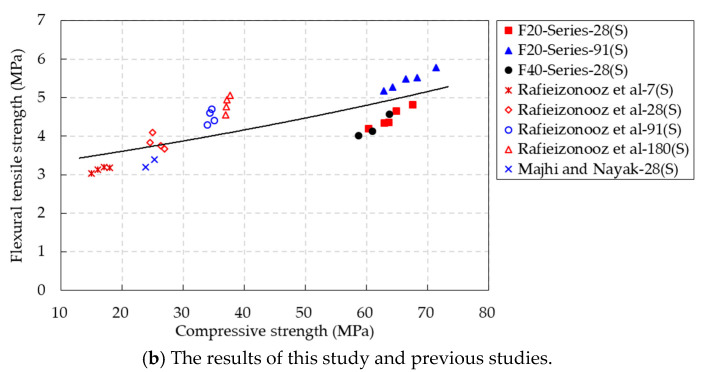
Relationship between the flexural tensile strength and compressive strength of the CBA concrete.

The relationship between the flexural tensile strength and compressive strength of the CBA concrete under the SSD conditions is presented in Figure 12a. An exponential equation for predicting the flexural tensile strength of the CBA concrete by using the compressive strength was suggested as follows:

(6) $$f_{r,\mathrm{SSD}}=0.7098e^{0.0296f_{c,\mathrm{SSD}}^{\prime }}\;R^{2}=0.7056\;(\mathrm{SSD}\;\mathrm{conditions})$$

where *f_r,_*_SSD_ is the flexural tensile strength (MPa) and *f′_c,_*_SSD_ is the compressive strength (MPa).

The measurements were scattered because they were measured at curing ages of 28 and 91 days. Therefore, the coefficient of determination of this equation was slightly low.

In addition, based on the test results in this study and previous studies [30,31], the relationship between the flexural tensile strength and compressive strength of the CBA concrete is presented in Figure 12b. The proposed equation for predicting the flexural tensile strength of CBA concrete is shown as follows:

(7) $$f_{r,\mathrm{SSD}}=3.1274e^{0.0071f_{c,\mathrm{SSD}}^{\prime }}\;R^{2}=0.5759\;(\mathrm{SSD}\;\mathrm{conditions})$$

where *f_r,_*_SSD_ is the flexural tensile strength (MPa) and *f′_c,_*_SSD_ is the compressive strength (MPa) under the SSD conditions. This equation showed a low coefficient of determination with a value of 0.5759 because most test results deviated from the prediction line. It was assumed that the accuracy of prediction would be enhanced if more test results were included.

**Figure 13:**
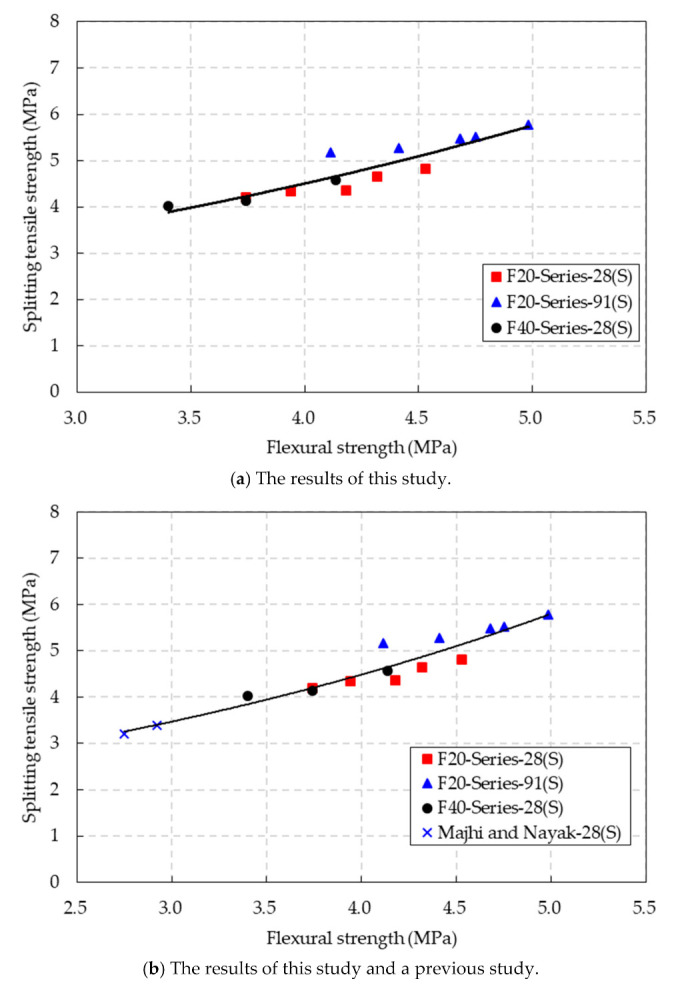
Relationship between the splitting tensile strength and flexural strength of the CBA concrete.

Figure 13a shows the relationship between the splitting tensile strength and flexural tensile strength of the CBA concrete under the SSD conditions. Based on the regression analysis, an exponential equation to relate the splitting tensile strength of the CBA concrete to the flexural tensile strength was proposed as follows:

(8) $$f_{t,\mathrm{SSD}}=1.6841e^{0.246f_{r,\mathrm{SSD}}}\;R^{2}=0.8272\;(\mathrm{SSD}\;\mathrm{conditions})$$

where *f_t,_*_SSD_ is the splitting tensile strength (MPa) and *f_r,_*_SSD_ is the flexural tensile strength (MPa).

In addition, the relationship between the splitting tensile strength and flexural tensile strength of the CBA concrete, based on the test results in this study and a previous study [30], is presented in Figure 13b. The equation for predicting the splitting tensile strength through the flexural tensile strength of the CBA concrete was proposed as follows:

(9) $$f_{t,\mathrm{SSD}}=1.607e^{0.2567f_{r,\mathrm{SSD}}^{}}\;R^{2}=0.9238\;(\mathrm{SSD}\;\mathrm{conditions})$$

where *f_t,_*_SSD_ is the splitting tensile strength (MPa) and *f_r,_*_SSD_ is the flexural tensile strength (MPa) under the SSD conditions. This equation presents a high coefficient of determination of 0.9238. This finding indicates that the proposed equation could be practically used to predict the splitting tensile strength of CBA concrete based on the flexural tensile strength.

The strength of the CBA concrete might be decreased by CBA as a partial or full replacement of aggregate. In particular, the use of concrete containing CBA as a replacement for coarse aggregate is hardly used in structural members due to high strength reduction but is usually used in nonstructural members of concrete structures.

Meanwhile, the strength test results in this study showed that concrete containing CBA as a replacement of fine aggregate and fly ash as a partial replacement of cement obtained remarkable strength of the concrete. Moreover, due to the pozzolanic reaction of fly ash, the replacement of fine aggregate with fly ash could overcome the reduction of strength in the CBA concrete in the long term. Finally, the overall strength test results in this study implied that concrete containing CBA as a partial replacement of fine aggregate could be used in structural members if the strength reduction due to CBA fine aggregate was acceptable in terms of the design of reinforced concrete structures.

However, the investigation of this study was restricted to selected mechanical properties, such as compressive, splitting tensile, and flexural tensile strength. To extend the use of the concrete analyzed in this study, more characteristics of concrete, for example, durability and bonding of the concrete, should be analyzed, but they were out of the scope of this study.

### 4.7. Ultrasonic Velocity

**Figure 14:**
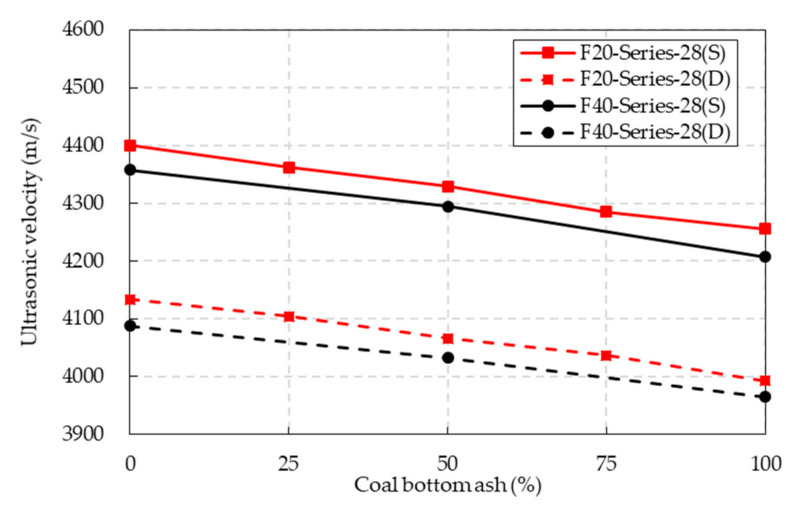
Ultrasonic velocity of the CBA concrete.

The test results of the ultrasonic pulse velocity of CBA concrete are shown in Figure 14 and Table 6. The ultrasonic pulse velocity of the CBA concrete decreased as the CBA content increased under both the SSD and oven-dried conditions. Moreover, the drying condition significantly affected the ultrasonic pulse velocity of the CBA concrete. The ultrasonic velocities of both the F20 and F40 series were considerably lower under the oven-dried conditions than under the SSD conditions. The free water in the CBA concrete evaporated after the concrete was oven-dried for 24 h at approximately 105 °C, which caused a reduction in the ultrasonic velocity.

### 4.8. Relationships between the Ultrasonic Velocity and Compressive, Splitting Tensile, and Flexural Tensile Strengths

The relationships between the ultrasonic velocity and compressive and splitting tensile strengths of concrete containing CBA aggregate were suggested in some studies [16,17,31], but the concrete investigated in this study incorporated fly ash and CBA aggregate. In addition, previous studies did not usually consider the relationships between the ultrasonic velocity and strength of CBA concrete under different curing and drying conditions. However, the relationship between the ultrasonic velocity and strength of concrete under different curing and drying conditions is suggested in this study.

**Figure 15:**
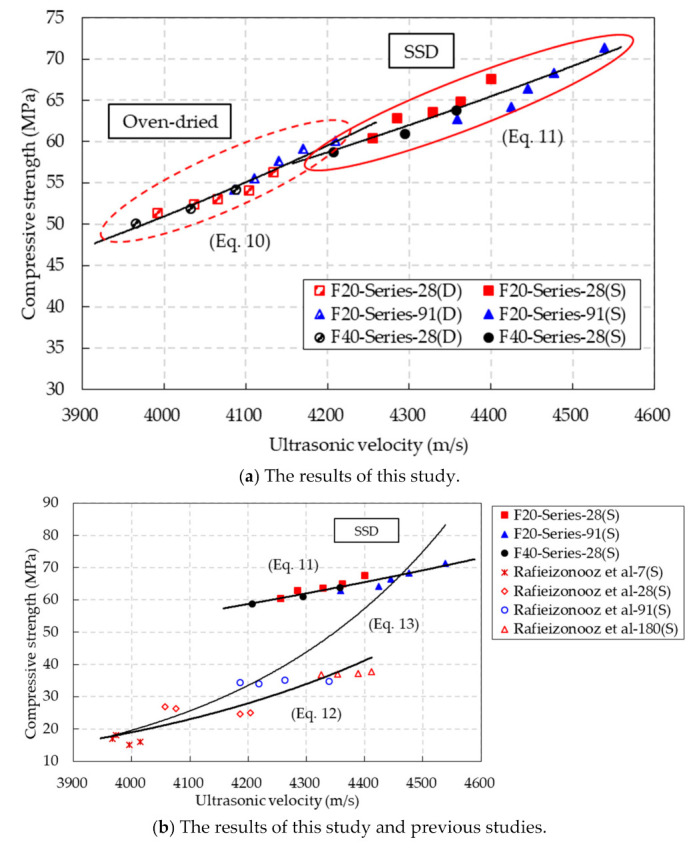
Relationships between the ultrasonic velocity and compressive strength under SSD conditions.

The relationship between the compressive strength and ultrasonic velocity under the SSD and oven-dried conditions is presented in Figure 15a. The test results showed that the compressive strength increased with the ultrasonic velocity of the CBA concrete under both the SSD and oven-dried conditions. By regression analysis, the relations between the compressive strength and ultrasonic velocity under oven-dried and SSD conditions were expressed by the following equation:

(10) $$f_{c,\mathrm{OD}}^{\prime }=2.2669e^{0.0008V_{\mathrm{OD}}}\;R^{2}=0.9564\;(\mathrm{Oven}-\mathrm{dried}\;\mathrm{conditions})$$

(11) $$f_{c,\mathrm{SSD}}^{\prime }=5.9323e^{0.0005V_{\mathrm{SSD}}}\;R^{2}=0.8956\;(\mathrm{SSD}\;\mathrm{conditions})$$

where *f′_c,_*_OD_ and *f′_c,_*_SSD_ are the compressive strengths (MPa) under oven-dried and SSD conditions, respectively. *V*_OD_ and *V*_SSD_ are the ultrasonic velocity (m/s) under oven-dried and SSD conditions, respectively. Regression analysis indicated that the coefficient of determination of the equation was close to 1, which indicates that this equation could be effectively used to predict the compressive strength.

Meanwhile, Rafieizonooz et al. [31] suggested an equation to predict the compressive strength by using the ultrasonic velocity as follows:

(12) $$f_{c,\mathrm{SSD}}^{\prime }=0.0111e^{1.8593V_{\mathrm{SSD}}}$$

The test results obtained from this study and previous studies are shown in Figure 15b, and the comparison of the predictions by using Equations (11) and (12) is also shown in the figure. Predictions by Equation (11) based on this study were not in good agreement with the previous test results, while predictions by Equation (12) based on the previous study were not in good agreement with the test results in this study. These findings occurred because Equations (11) and (12) were proposed based on their individual test results.

Therefore, based on the test results in this study and previous studies, an additional exponential equation for predicting the compressive strength of the CBA concrete was proposed as follows:

(13) $$f_{c,\mathrm{SSD}}^{\prime }=0.0004e^{0.0027V_{\mathrm{SSD}}}$$

Moreover, to estimate the level of prediction accuracy, the sum of squared errors for the prediction equation was calculated as follows:

(14) $$\Delta^{2}=\sum_{i}^{n}(y_{i}-y_{i}^{\prime }{)}^{2}$$

where Δ^2^ is the sum of the squared errors, *y_i_* is the measurement, and *y*′*_i_* is the prediction.

The sums of the squared errors (Δ^2^) for Equations (11)–(13) were 8432, 9628, and 4319, respectively. This comparison showed that the sum of the squared errors in Equation (13) was the lowest among the three equations. Therefore, compared with Equations (11) and (12), Equation (13) more accurately predicted the compressive strength of the CBA concrete by using the ultrasonic velocity.

**Figure 16:**
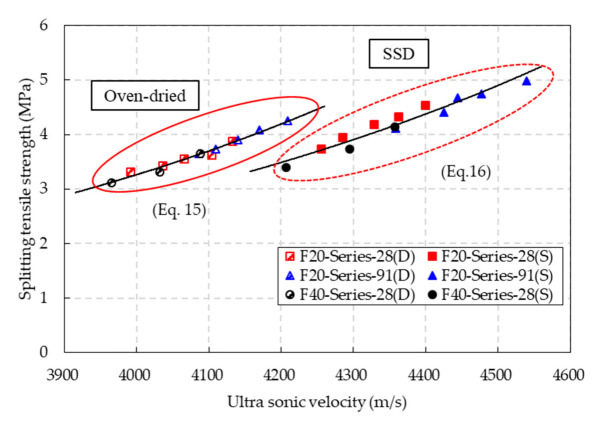
Relationships between the ultrasonic velocity and splitting tensile strength under SSD and oven-dried conditions.

The relationships between the splitting tensile strength and ultrasonic velocity of the CBA concrete are presented in Figure 16. Two exponential equations for predicting the splitting tensile strength of the CBA concrete under oven-dried and SSD conditions by using the ultrasonic velocity were suggested as follows:

(15) $$f_{t,\mathrm{OD}}=0.0218e^{0.0013V_{\mathrm{OD}}}\;R^{2}=0.9765\;(\mathrm{Oven}-\mathrm{dried}\;\mathrm{conditions})$$

(16) $$f_{t,\mathrm{SSD}}=0.0297e^{0.0011V_{\mathrm{SSD}}}\;R^{2}=0.9415\;(\mathrm{SSD}\;\mathrm{conditions})$$

where *f_t,_*_OD_ and *f_t,_*_SSD_ are the compressive strengths (MPa) under oven-dried and SSD conditions, respectively. *V*_OD_ and *V*_SSD_ are the ultrasonic velocity (m/s) under oven-dried and SSD conditions, respectively. These equations predicted the splitting tensile strength of the CBA concrete well.

**Figure 17:**
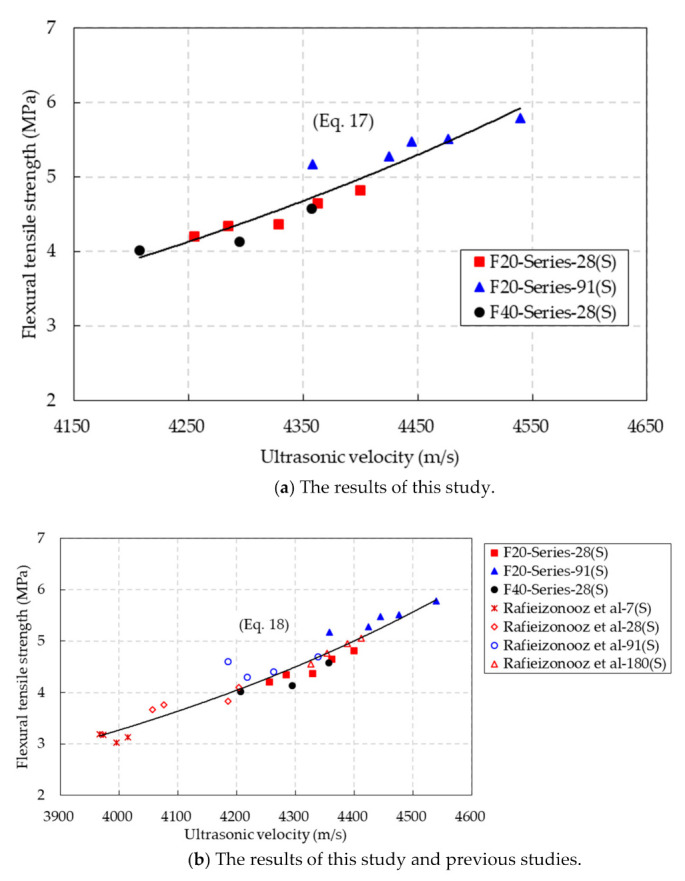
Relationship between the ultrasonic velocity and flexural tensile strength under SSD conditions.

Figure 17a shows the relationship between the flexural tensile strength and ultrasonic velocity of the CBA concrete under the SSD conditions. An exponential equation for predicting the flexural tensile strength of CBA concrete by using the ultrasonic velocity was proposed as follows:

(17) $$f_{r,\mathrm{SSD}}=0.0209e^{0.0012V_{\mathrm{SSD}}}\;R^{2}=0.8931\;(\mathrm{SSD}\;\mathrm{conditions})$$

As described in the regression equation to predict compressive strength, the coefficient of determination of the proposed equation for the flexural tensile strength was close to 1.

In addition, the relationship between the ultrasonic velocity and flexural strength of the CBA concrete, based on the test results in this study and a previous study [31], is shown in Figure 17b. Based on the test results in this study and previous studies, exponential equations for predicting the flexural tensile strength through the ultrasonic velocity of CBA concrete were proposed as follows:

(18) $$f_{r,\mathrm{SSD}}^{}=0.0463e^{0.0011V_{\mathrm{SSD}}}\;R^{2}=0.9243\;(\mathrm{SSD}\;\mathrm{conditions})$$

The predictions in Figure 17b show that the experimental results were close to the predicted results, and thus, the proposed equation had a high coefficient of determination of 0.9243. Moreover, the sum of the squared errors obtained for Equation (18) at 16.89 was lower than that for Equation (17) at 22.11. These findings indicate that the suggested equation, which was based on the test results in this study and a previous study, could accurately predict the flexural tensile strength of the CBA concrete by using the ultrasonic velocity.

## 5. Conclusions

The effects of the curing and drying conditions on the strength properties of CBA concrete at various CBA contents were investigated in this study. Given the extensive experimental results, the following conclusions could be drawn:

1. The unit weight of the CBA concrete containing fly ash was sensitive to oven-drying. Specifically, the unit weight of the CBA concrete was reduced by approximately 5% when the drying method was changed from the SSD conditions to oven-dried conditions. The reduction in the unit weight resulted from the evaporation of the absorbed water and free water in the CBA concrete specimens. In addition, the substitution of natural aggregates and cement with CBA and fly ash tended to reduce the unit weight of the CBA concrete by approximately 4.8~5.7%.
2. The compressive and splitting tensile strengths of the CBA concrete decreased significantly under oven-dried conditions. The compressive and splitting tensile strengths under oven-dried conditions were approximately 15% lower than those under SSD conditions. This implied that they depended on the drying condition of the concrete specimen. However, the CBA and fly ash contents slightly contributed to the reduction in the compressive and splitting tensile strengths of the CBA concrete.
3. The flexural tensile strength of the CBA concrete was also slightly influenced by the replacement of fine aggregate with CBA. However, the flexural tensile strength of the CBA concrete was considerably enhanced at a long-term curing age by approximately 22%. The test results revealed that the flexural strength of the CBA concrete was increased by the pozzolanic reaction of the CBA and fly ash materials.
4. Strength test results implied that the replacement of fine aggregate with fly ash could overcome the reduction of strength in the CBA concrete in the long term due to the pozzolanic reaction of fly ash. Therefore, in terms of strength, concrete containing CBA as a replacement of fine aggregate and fly ash as a partial replacement of cement could be used in structural members.
5. The drying conditions caused a significant decrease in the ultrasonic velocity in the CBA concrete. For the F20 series concrete, the ultrasonic velocities under oven-dried conditions were 5.8~6.2% lower than those under SSD conditions with various CBA contents. For the F40 series concrete, the ultrasonic velocities under oven-dried conditions were 5.8~6.6% lower than those under SSD conditions with various CBA contents.
6. The relationships between the ultrasonic velocity and compressive, splitting tensile, and flexural tensile strengths under the SSD and oven-dried conditions were presented. The relationships highly varied depending on the drying and curing conditions. Therefore, when the strength of the CBA concrete is predicted by using ultrasonic velocity measurements, the drying and curing conditions should be considered.

## Figures and Tables

**Table 1:** Physical properties of aggregates used.

Aggregate	Density	Water Absorption	Fineness Modulus
	(g/cm^3^)	(%)	
Natural coarse aggregate	2.60	1.02	6.68
Natural fine aggregate	2.61	0.64	2.90
CBA	1.84	3.88	3.79

**Table 2:** Chemical compositions of OPC, CBA, and fly ash.

Component	OPC	CBA	Fly Ash
	(%)	(%)	(%)
SiO_2_	17.60	55.70	55.80
Al_2_O_3_	4.59	26.20	22.10
Fe_2_O_3_	3.43	7.53	9.26
CaO	65.00	3.93	6.42
Na_2_O	0.19	0.76	1.33
MgO	3.53	1.09	1.69
K_2_O	1.13	1.17	1.30
SO_3_	3.76	0.76	-
LOI	0.76	2.40	1.80

**Table 3:** Mixing proportions.

Mixture	FA Content by Volume (%)	CBA Content by Volume (%)	w/b	Unit Weight (kg/m^3^)							
				Water	Binder		Coarse Aggregate	Fine Aggregate		Admixture	
					OPC	FA		Natural	CBA	HWRA	AE
F20-B000-28 (91)	20	0	0.32	178.5	476.0	84.2	878.5	650.0	0.0	6.72	1.88
F20-B025-28 (91)		25	0.32	178.5	476.0	84.2	878.5	487.5	115.4	6.72	1.88
F20-B050-28 (91)		50	0.32	178.5	476.0	84.2	878.5	325.0	230.7	6.72	1.88
F20-B075-28 (91)		75	0.32	178.5	476.0	84.2	878.5	162.5	346.1	6.72	1.88
F20-B100-28 (91)		100	0.32	178.5	476.0	84.2	878.5	0.0	461.4	6.72	1.88
F40-B000-28	40	0	0.34	178.5	357.0	168.5	878.5	650.0	0.0	6.31	1.77
F40-B050-28		50	0.34	178.5	357.0	168.5	878.5	325.0	230.7	6.31	1.77
F40-B100-28		100	0.34	178.5	357.0	168.5	878.5	0.0	461.4	6.31	1.77

Notes: OPC: ordinary Portland cement; FA: fly ash; CBA: coal bottom ash; HWRA: high-range water-reducing agent; AE: air-entraining agent.

**Table 4:** Compressive strength and elastic modulus results.

Mixture	Compressive Strength (MPa)								Elastic Modulus (MPa)							
	28 Days				91 Days				28 Days				91 Days			
	SSD		Oven-Dried		SSD		Oven-Dried		SSD		Oven-Dried		SSD		Oven-Dried	
	Mean	S.D.	Mean	S.D.	Mean	S.D.	Mean	S.D.	Mean	S.D.	Mean	S.D.	Mean	S.D.	Mean	S.D.
F20-B000	67.6	6.0	56.3	6.4	71.4	5.5	60.1	3.7	24,990	1367	23,060	1092	27,340	2163	23,300	1354
F20-B025	64.9	5.7	54.0	8.8	68.3	3.6	59.1	11.3	24,840	1842	22,300	1250	26,160	1112	22,910	947
F20-B050	63.6	8.2	53.0	5.9	66.4	3.3	57.7	9.5	24,260	1690	22,000	1863	25,440	862	22,440	1895
F20-B075	62.9	7.1	52.4	7.9	64.2	6.8	55.6	5.2	23,840	985	21,440	1159	24,600	793	22,130	1045
F20-B100	60.4	6.1	51.3	6.8	62.8	4.7	54.2	9.6	23,260	1362	20,448	1766	24,040	1486	21,720	1762
F40-B000	63.8	8.7	54.1	7.6	-	-	-	-	23,550	1980	22,500	1228	-	-	-	-
F40-B050	61.0	5.5	51.9	6.4	-	-	-	-	22,990	1563	21,370	1595	-	-	-	-
F40-B100	58.7	5.3	50.1	4.4	-	-	-	-	22,450	1485	20,000	990	-	-	-	-

Notes: S.D.: standard deviation.

**Table 5:** Splitting and flexural tensile strength results.

Mixture	Splitting Tensile Strength (MPa)								Flexural Tensile Strength (MPa)			
	28 Days				91 Days				28 Days		91 Days	
	SSD		Oven-Dried		SSD		Oven-Dried		SSD		SSD	
	Mean	S.D.	Mean	S.D.	Mean	S.D.	Mean	S.D.	Mean	S.D.	Mean	S.D.
F20-B000	4.53	0.52	3.87	0.15	4.98	0.21	4.26	0.14	4.82	0.41	5.79	0.60
F20-B025	4.32	0.24	3.62	0.36	4.75	0.35	4.08	0.86	4.65	0.92	5.52	0.62
F20-B050	4.18	0.86	3.55	0.16	4.68	0.83	3.91	0.97	4.36	0.41	5.48	0.08
F20-B075	3.94	0.13	3.42	0.29	4.41	0.34	3.74	0.34	4.34	0.76	5.28	0.53
F20-B100	3.74	0.87	3.32	0.09	4.11	0.92	3.65	0.48	4.20	0.93	5.17	0.23
F40-B000	4.14	0.09	3.65	0.15	-	-	-	-	4.58	0.58	-	-
F40-B050	3.74	0.68	3.32	0.07	-	-	-	-	4.14	0.12	-	-
F40-B100	3.40	0.45	3.12	0.08	-	-	-	-	4.02	0.27	-	-

Notes: S.D.: standard deviation.

**Table 6:** Ultrasonic velocity results.

Mixture	Ultrasonic Velocity (mm/s)							
	28 Days				91 Days			
	SSD		Oven-Dried		SSD		Oven-Dried	
	Mean	S.D.	Mean	S.D.	Mean	S.D.	Mean	S.D.
F20-B000	4400	41	4133	37	4539	54	4209	44
F20-B025	4362	39	4104	21	4477	27	4170	32
F20-B050	4329	34	4066	40	4445	31	4140	19
F20-B075	4285	29	4037	15	4425	29	4110	28
F20-B100	4255	28	3992	46	4358	32	4086	36
F40-B000	4357	17	4088	23	-	-	-	-
F40-B050	4295	48	4033	17	-	-	-	-
F40-B100-	4207	20	3965	36	-	-	-	-

Notes: S.D.: standard deviation.

## Data Availability

The data used to support the findings in this study are available from the corresponding author upon request.
